# 842. Histoplasmosis : An observational study from northern India in non-HIV population.

**DOI:** 10.1093/ofid/ofad500.887

**Published:** 2023-11-27

**Authors:** Satish Swain, Saurav Sekhar Paul, Adarsh Aayilliath, Sayan Maharatna, Bhavesh Mohan Lal, Sryla Punjadath, Imtiyaz Shareef, Gagandeep Singh, Immaculata Xess, Manish Soneja, Naveet Wig

**Affiliations:** All India Institute of Medical Sciences, New Delhi, NEW DELHI, Delhi, India; All india Institute of Medical Sciences, New Delhi, delhi, Delhi, India; All India Institute of Medical Sciences, New Delhi, New Delhi, Delhi, India; All India Institute of Medical Sciences, New Delhi, NEW DELHI, Delhi, India; All India Institute of Medical Sciences, New Delhi, NEW DELHI, Delhi, India; All India Institute of Medical Sciences, New Delhi, NEW DELHI, Delhi, India; All India Institute of Medical Sciences, New Delhi, NEW DELHI, Delhi, India; All India Institute of Medical Sciences, New Delhi, NEW DELHI, Delhi, India; All India Institute of Medical Sciences, New Delhi, NEW DELHI, Delhi, India; All India Institute Of Medical Sciences, Delhi, Delhi, India; All India Institute of Medical Sciences, DELHI, Delhi, India

## Abstract

**Background:**

Globally around 40% of histoplasmosis has been reported in HIV population, with other risk factors being transplant recipients, immunosuppressive agents (steroids, TNF-alpha inhibitors) and extreme of ages. Histoplasmosis in India has mostly been reported from the Gangetic plains (Figure 1).

**Figure:**
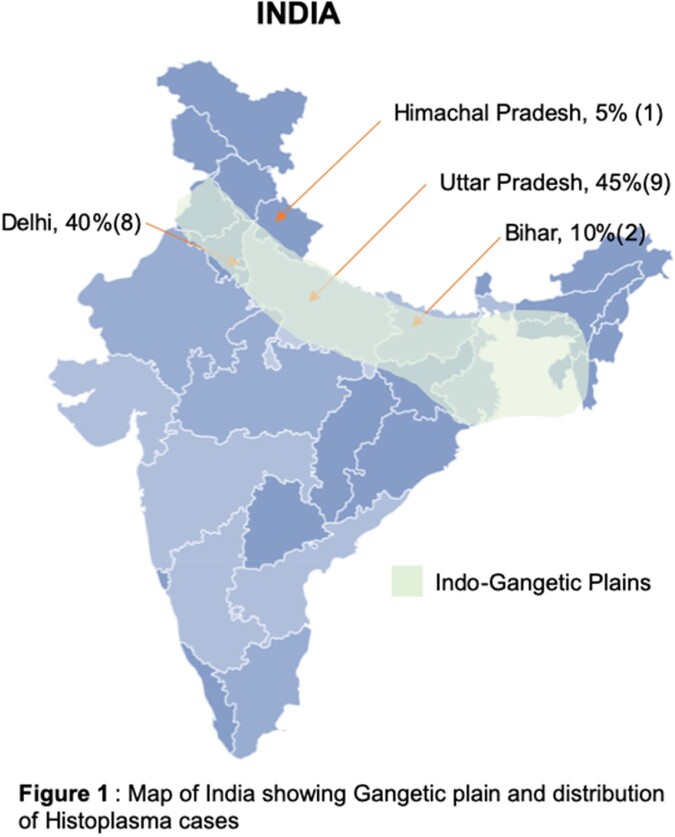

**Methods:**

This current study was conducted in a tertiary care hospital of northern India between January 1, 2021 and December 31, 2022 to look at the epidemiology, clinical profile and treatment outcome of Histoplasmosis patients in a non-HIV cohort. This was a single centered, observational study. All patients with proven Histoplasmosis (according to EORTC/MSGERC 2019 criteria) were included.

**Results:**

*Baseline Characteristics*: This study involved 20 patients with a mean age of 52.05 ± 10.87 years with 70% being male. All the patients were from endemic areas of Gangetic belt in India. Majority of the patients (80%) were immunocompetent (Table 1). All cases (100%) were diagnosed on histopathology.

**Clinical and Laboratory Characteristics:**

The common reported complains were fever (75%), weight loss (70%), loss of appetite (75%), skin lesions (25%) and pain abdomen (15%). Mean duration of various symptoms was 3±1.29 months. Hepato-splenomegaly was seen in 45% of cases followed by adrenal involvement (40%), enlarged lymph nodes (35%), skin and oral mucosa involvement (25%) (Figure 2). Out eight patients had who adrenal involvement, five patients (25%) had isolated involvement based on imaging (CT/PET-CT). Cytopenias was seen in upto 75% cases with around 40% had deranged liver functions (Table 2). The most common syndromic diagnosis was progressive disseminated histoplasmosis (PDH) seen in 13 patients with a median age of 49 (30-76) years.

**Treatment:**

13 patients (65%) received induction with Amphotericin B (liposomal) followed by switching to oral itraconazole and 7 patients (35%) got up front itraconazole. At a media follow up of 8 months, 40% of patients had completed treatment and one had patient died.

**Figure:**
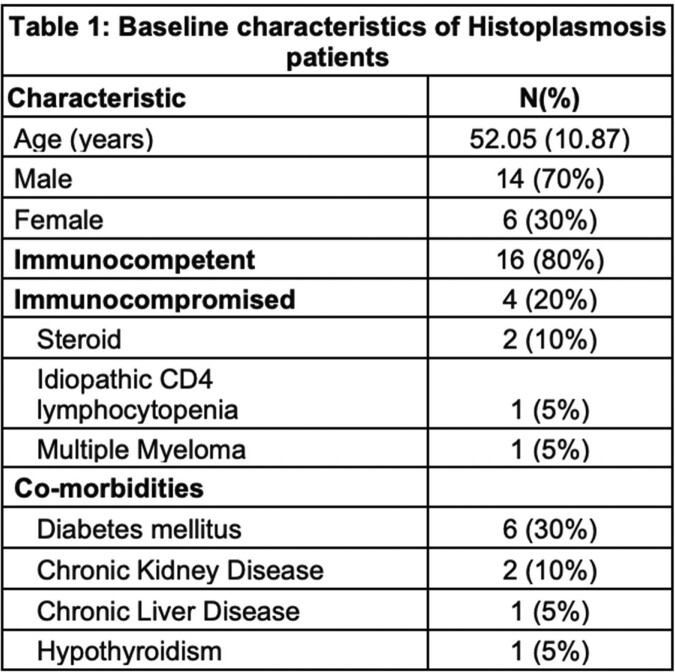

**Figure:**
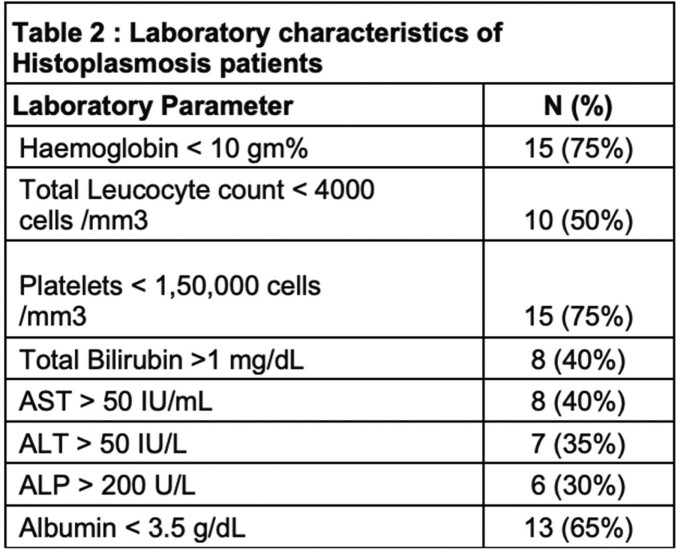

**Figure:**
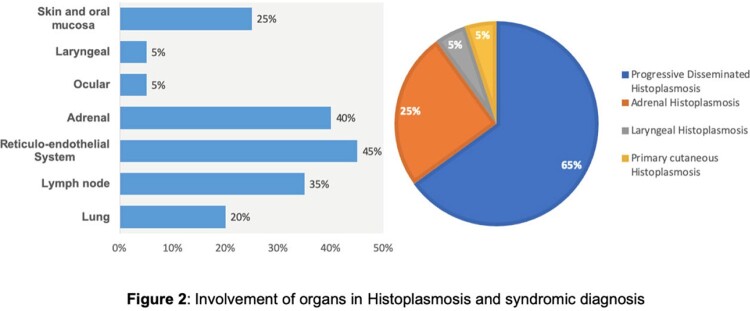

**Conclusion:**

In a high tuberculosis endemic county like India, Histoplasmosis pose a diagnostic challenge. Although the median time to diagnosis is longer (delay in diagnosis), the overall outcome is good. Majority of our patients were younger immunocompetent individual.

**Disclosures:**

**All Authors**: No reported disclosures

